# Deep learning super-resolution reconstruction for fast and high-quality cine cardiovascular magnetic resonance

**DOI:** 10.1007/s00330-024-11145-0

**Published:** 2024-10-23

**Authors:** Dmitrij Kravchenko, Alexander Isaak, Narine Mesropyan, Johannes M. Peeters, Daniel Kuetting, Claus C. Pieper, Christoph Katemann, Ulrike Attenberger, Tilman Emrich, Akos Varga-Szemes, Julian A. Luetkens

**Affiliations:** 1 Department of Diagnostic and Interventional Radiology, University Hospital Bonn, Bonn, Germany; 2 Quantitative Imaging Laboratory Bonn, Bonn, Germany; 3 Division of Cardiovascular Imaging, Department of Radiology and Radiological Science, Medical University of South Carolina, Charleston, SC USA; 4 Philips MR Clinical Science, Best, Netherlands; 5 Philips GmbH Market DACH, Hamburg, Germany; 6 Department of Diagnostic and Interventional Radiology, University Medical Center of the Johannes Gutenberg-University, Mainz, Germany; 7 German Centre for Cardiovascular Research, Partner site Rhine-Main, Mainz, Germany

**Keywords:** Cardiovascular, Magnetic resonance imaging (cine), Artificial intelligence (AI)

## Abstract

**Objectives:**

To compare standard-resolution balanced steady-state free precession (bSSFP) cine images with cine images acquired at low resolution but reconstructed with a deep learning (DL) super-resolution algorithm.

**Materials and methods:**

Cine cardiovascular magnetic resonance (CMR) datasets (short-axis and 4-chamber views) were prospectively acquired in healthy volunteers and patients at normal (cine_NR_: 1.89 × 1.96 mm^2^, reconstructed at 1.04 × 1.04 mm^2^) and at a low-resolution (2.98 × 3.00 mm^2^, reconstructed at 1.04 × 1.04 mm^2^). Low-resolution images were reconstructed using compressed sensing DL denoising and resolution upscaling (cine_DL_). Left ventricular ejection fraction (LVEF), end-diastolic volume index (LVEDVi), and strain were assessed. Apparent signal-to-noise (aSNR) and contrast-to-noise ratios (aCNR) were calculated. Subjective image quality was assessed on a 5-point Likert scale. Student’s paired *t*-test, Wilcoxon matched-pairs signed-rank-test, and intraclass correlation coefficient (ICC) were used for statistical analysis.

**Results:**

Thirty participants were analyzed (37 ± 16 years; 20 healthy volunteers and 10 patients). Short-axis views whole-stack acquisition duration of cine_DL_ was shorter than cine_NR_ (57.5 ± 8.7 vs 98.7 ± 12.4 s; *p* < 0.0001). No differences were noted for: LVEF (59 ± 7 vs 59 ± 7%; ICC: 0.95 [95% confidence interval: 0.94, 0.99]; *p* = 0.17), LVEDVi (85.0 ± 13.5 vs 84.4 ± 13.7 mL/m^2^; ICC: 0.99 [0.98, 0.99]; *p* = 0.12), longitudinal strain (−19.5 ± 4.3 vs −19.8 ± 3.9%; ICC: 0.94 [0.88, 0.97]; *p* = 0.52), short-axis aSNR (81 ± 49 vs 69 ± 38; *p* = 0.32), aCNR (53 ± 31 vs 45 ± 27; *p* = 0.33), or subjective image quality (5.0 [IQR 4.9, 5.0] vs 5.0 [IQR 4.7, 5.0]; *p* = 0.99).

**Conclusion:**

Deep-learning reconstruction of cine images acquired at a lower spatial resolution led to a decrease in acquisition times of 42% with shorter breath-holds without affecting volumetric results or image quality.

**Key Points:**

***Question*** *Cine CMR acquisitions are time-intensive and vulnerable to artifacts*.

***Findings*** *Low-resolution upscaled reconstructions using DL super-resolution decreased acquisition times by 35–42% without a significant difference in volumetric results or subjective image quality*.

***Clinical relevance*** *DL super-resolution reconstructions of bSSFP cine images acquired at a lower spatial resolution reduce acquisition times while preserving diagnostic accuracy, improving the clinical feasibility of cine imaging by decreasing breath hold duration*.

**Graphical Abstract:**

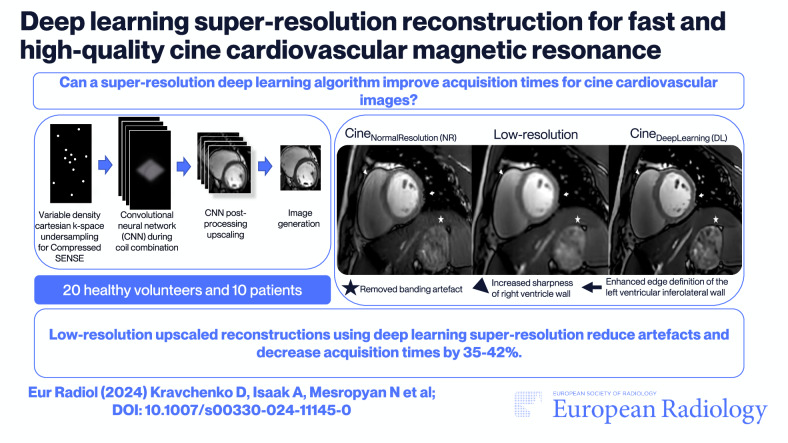

## Introduction

Cardiovascular magnetic resonance (CMR) is employed for the diagnosis of various cardiac pathologies such as acute myocarditis, myocardial infarction, or cardiomyopathies. Cine CMR is, in particular, the backbone of most CMR investigations, providing dynamic information pertaining to cardiac anatomy and function [1]. Unfortunately, CMR examinations have relatively long acquisition times due to the high volume of data required for adequate temporal and spatial resolution reconstructions, contributing to patient discomfort and having negative economic implications [2]. Under ideal settings, a standard CMR examination for the exclusion of myocarditis or myocardial infarction takes around 45 min, with many instances of breath holds required for image acquisition. Alone, short-axis cine CMR acquisitions consist of 12–15 breath holds, and can each take up to 15 s to acquire with an additional 10 s for recovery after each breath hold, for a total of up to 5 min per stack [3–5]. Limited breath-holding capacity, for example in multimorbid patients with heart failure, sedated patients, or in children with congenital heart disease, often leads to difficult examination conditions and artifact-prone acquisitions [6–9]. Cardiovascular imagers are tasked with balancing the need for shorter breath holds to avoid respiratory motion and the required time to acquire images with sufficient spatial and temporal resolution needed for cardiac assessment. Some techniques, such as free breathing allow for stable image acquisitions but tend to take longer than routinely used breath hold methods [3, 10]. The optimization of already routinely used CMR sequences and the development of novel imaging techniques offer two approaches to address the issue of CMR scan times. Recent advancements in imaging techniques such as the application of compressed sensing [11, 12] in CMR cine acquisitions and deep learning (DL) algorithm reconstructions of under-sampled, low-resolution acquisitions have already demonstrated some potential clinical applications in other fields such as MR prostate imaging [7, 13, 14]. However, currently, there is a paucity of data comparing existing standard cine acquisitions to DL-assisted reconstructions.

The aim of this study was to compare standard balanced steady-state free precession (bSSFP) cine images with low-resolution bSSFP cine images reconstructed with a DL super-resolution (SR) algorithm.

## Materials and methods

This cross-sectional, single-center study with a prospectively acquired study cohort was performed in concordance with the Declaration of Helsinki and the International Conference on Harmonization of Good Clinical Practice. Study design, information processing, and implementation were approved by the institutional review board. All participants (healthy volunteers and patients) gave written consent before inclusion in the study.

### Study participants

Prospective random enrollment of patients with clinical indications for contrast-enhanced CMR occurred between November 2022 and February 2023. Additionally, healthy volunteers over the age of 18 years old without any known cardiac diseases were prospectively recruited. Inclusion criteria were: age over 18 years old, able to give consent. Exclusion criteria were: pregnancy, implementation of cardiac pacemakers, or other contraindications for examinations on 3.0-T MRI scanners.

### CMR protocol and normal resolution reconstructions

All sequences were acquired using a 3.0-T scanner (Philips Ingenia 3.0-T; Philips Healthcare) using a 16-channel torso coil with a digital interface. Patients underwent a routine CMR protocol comprised of electrocardiogram-triggered bSSFP cine imaging acquired at normal resolution (cine_NR_) in short-axis views (field of view: 250 × 250 mm^2^, repetition time: 3.1 ms, echo time: 1.54 ms, flip angle: 45°, in-plane resolution: 1.89 × 1.96 mm^2^ [reconstructed: 1.04 × 1.04 mm^2^], slice thickness: 8 mm, temporal resolution: 45 ms, compressed sensitivity encoding (Compressed SENSE) factor: 2.5), 4-chamber views, 2-chamber views, and 3-chamber views. Additionally, patients received a standard of care protocol consisting of T2 short-tau inversion recovery sequences, T1 and T2 mapping, and segmented inversion-recovery gradient-echo sequences for late gadolinium enhancement (LGE) using the Look-Locker method [15] after intravenous contrast injection (0.2 mmol/kg of body weight bolus of gadoterate meglumine [Clariscan; GE Healthcare]). For study purposes, healthy volunteers and patients (in addition to the standard of care imaging after contrast injection), underwent an electrocardiogram-triggered low-resolution bSSFP cine was acquisition (cine_DL_) in short-axis views (field of view: 250 × 250 mm^2^, repetition time: 2.9 ms, echo time: 1.34 ms, flip angle: 45°, in-plane resolution: 2.98 × 3.00 mm^2^ [reconstructed: 1.04 × 1.04 mm^2^], slice thickness: 8 mm, temporal resolution: 45 ms, Compressed SENSE factor: 2.5) and 4-chamber views, which were acquired after the cine_NR_ sequences and reconstructed with an SR DL algorithm.

### DL image reconstruction

Images were reconstructed using a vendor-provided prototype (Philips NGSA patch). Only non-industry personnel had full access to all acquired study data. A series of convolutional neural networks (CNNs) were applied to the raw low-resolution *k*-space acquisitions as previously described [14]. The Aadaptive-CS-Net facilitated sparsity-constrained reconstruction of acquired images with Compressed SENSE-based variable density under-sampling patterns [16–18] and was applied during coil combination, removing noise and under-sampling artifacts [19]. Adaptive-CS-Net integrated multiscale sparsification with a CNN-based sparsifying approach with image reconstruction of Compressed SENSE, ensuring data consistency. Domain-specific knowledge such as image background location and coil sensitivity distribution was also incorporated to replace the usual process of wavelet transformation. A multilayer approach outputs each scale transformation consisting of 2D convolutional rectifier layers and a maximum pooling layer for downscaling or a bilinear interpolation for upscaling to down- or upscale transforms via direct and skip connections. Regularization optimization was performed by trained threshold levels for each connection. The Adaptive-CS-Net was pretrained on about 740,000 pairs of images of various contrasts and subsampling levels with applied supervised learning. The final output is a de-aliased, denoised MR image with preserved magnitude and phase. Subsequently, a second CNN, Precise Image Net, was applied to remove ringing artifacts and to replace the traditional zero-filling strategy to increase the matrix size and therewith the sharpness of the images [20, 21]. The combination of these CNNs made up the SR network [22, 23]. The network was trained on over six million pairs of low- and high-resolution data with *k*-space crops to induce ringing. This was achieved by repeatedly cascading a pair of 2D convolutional and rectifier layers ending with a data consistency check. Supervised learning was performed: for each image, a high-resolution version was downscaled to a lower-resolution image with truncation artifacts. Data consistency checks were implemented to match the resulting *k*-space with the measured *k*-space data. This study utilized a moderate level of noise reduction. Reconstructions were performed on scanner hardware equipped with an Nvidia Quadro RTX5000 GPU.

### Objective image analysis

Objective image analysis was performed by two board-certified cardiovascular radiologists (J.A.L. with 12 years of experience in CMR and D.Kr. with 5 years of experience in CMR) using dedicated software (IntelliSpace Portal, version 12.1.4; Philips Medical Systems). Left ventricular ejection fraction (LVEF), left ventricular end-diastolic volume index (LVEDVi), and interventricular septum thickness at diastole (IVSD) were measured in both groups. Apparent signal-to-noise ratios (aSNR) and apparent contrast-to-noise ratios (aCNR) were calculated as previously described [14]. Myocardial global systolic longitudinal, circumferential, and radial strain were calculated by using automatic feature tracking strain analysis software (Medis Suite MR, version 4.0.62.4, Medis Medical Imaging Systems) with manual corrections when necessary. Since cine_DL_ was only applied to 4-chamber and short-axis views, the global longitudinal strain was calculated from 4-chamber views for both cine_NR_ and cine_DL_.

### Subjective image analysis

Subjective image quality analysis was performed by two board-certified cardiovascular radiologists (D.Kr. with 5 years and A.I. with 6 years of experience in CMR). Subjective image quality was rated for cine_NR_ and cine_DL_ short-axis and 4-chamber views on a 5-point Likert scale regarding three image criteria: blood-pool to myocardium contrast, endocardial edge definition, and artifacts, as previously described [3, 24]. Raters were blinded and sequences were presented in random order. A total score was determined by the equal weight average of all three criteria:

- 1. Non-diagnostic: poor contrast between blood pool and myocardium, endocardial edge poorly defined, and artifacts render the images non-diagnostic.
- 2. Poor: blood pool barely discernable from the myocardium, washed-out endocardial edge, and blurring of trabeculae and numerous artifacts.
- 3. Adequate: blood pool discernable from myocardium but features lots of noticeable variation throughout the cardiac cycle, barely distinguishable endocardial edge definition, and some artifacts are present.
- 4. Good: the blood pool is mostly brighter and discernable from the myocardium, papillary and endocardial trabeculae are discernable but blurred in some images during the cardiac cycle, few artifacts are present but do not hinder image quality.
- 5. Excellent: the blood pool is hyperintense and clearly discernable from the myocardium in all images, and papillary and endocardial trabeculae are clearly visible with no blurring, and almost no artifacts.

### Statistical analysis

Statistical analysis was performed using Prism (version 10.1.0; GraphPad Software) and SPSS (version 29; IBM). Continuous variables for quantitative measurements are reported as means ± standard deviation (SD) and nominal data as percent to absolute frequency. The Shapiro–Wilk test was used to check for normality. Pearson’s correlation was used to compare the correlation between cine_NR_ and cine_DL_ volumetry results, aSNR, and aCNR. Median and interquartile range (IQR) are provided for nonparametric data or when normality cannot be assumed. Volumetric findings and acquisition times were compared using the paired Student’s *t*-test. The chi-squared test was used for nominal data comparisons. Subjective image scores were compared using the Wilcoxon matched-pairs signed rank test. Inter-rater agreement for subjective image quality, aSNR, aCNR, strain, and volumetry was compared using a two-way mixed effects intraclass correlation coefficient (ICC) model for absolute agreement. ICC was rated as poor (less than 0.5), moderate (0.5–0.75), good (0.75–0.9), and excellent (greater than 0.90) [25]. The level of statistical significance was set to *p* < 0.05.

## Results

### Participant characteristics

**Fig. 1:**
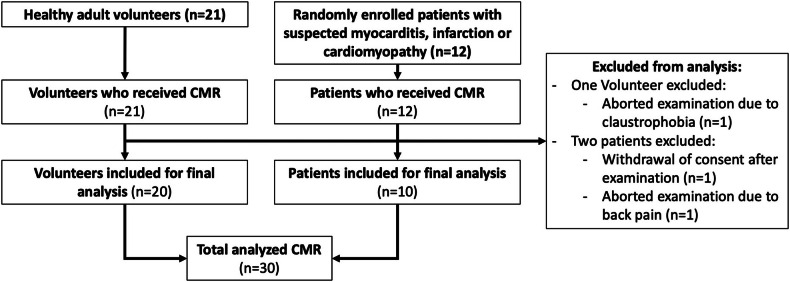
Flowchart depicting participant recruitment for the study

Overall, 20 healthy volunteers and 10 patients were included in the final analysis. In total, three participants had to be excluded (see study flow chart in Fig. 1). Participant characteristics are summarized in Table 1. Indications for CMR in the patient group were: suspicion of acute myocarditis (*n* = 1, 10%), aortic valve and subclavian artery stenosis (*n* = 1, 10%), heart failure of unknown cause (*n* = 1, 10%), Becker muscular dystrophy (*n* = 2, 20%), hypertrophic cardiomyopathy (*n* = 2, 20%), dilated cardiomyopathy (*n* = 1, 10%), and ischemic cardiomyopathy (*n* = 2, 20%).

**Table 1:** Clinical characteristics of participants

Groups/clinical parameters	All participants, (*n* = 30)	Healthy volunteers, (*n* = 20)	Patients, (*n* = 10)	*p* value
Age, (years)	37 ± 16	29 ± 3	53 ± 19	< 0.001
Sex, (males)	21 (70%)	14 (70%)	7 (70%)	0.99
Weight, (kg)	79 ± 18	77 ± 16	83 ± 20	0.22
Height, (cm)	174 ± 10	176 ± 8	171 ± 12	0.19
Body mass index, (kg/m^2^)	26 ± 5	25 ± 4	28 ± 6	0.24
Body surface area, (m^2^)	1.9 ± 0.2	1.9 ± 0.2	2.0 ± 0.3	0.29
Heart rate, (beats/min)	64 ± 11	65 ± 9	63 ± 14	0.03

**Fig. 2:**
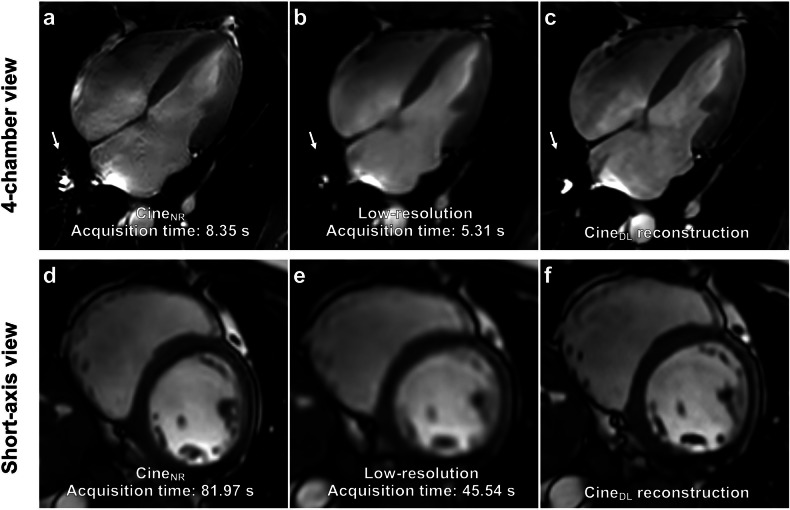
Four-chamber and short-axis views of a 31-year-old healthy male volunteer. Normal-resolution (cine_NR_) 4-chamber views (**a**) as well as the short-axis cine_NR_ (**d**) took longer to acquire than their low-resolution counterparts (**b**, **e**). The DL reconstructions (cine_DL_, **c**, **f**) from the low-resolution acquisitions resulted in a comparable image quality to the cine_NR_ images. The low-resolution acquisitions are noticeably blurrier compared to the cine_NR_ images. Notice the reduction in the pulsation artifact on the 4-chamber views going from the cine_NR_ to the low-resolution and cine_DL_ images (arrow)

**Fig. 3:**
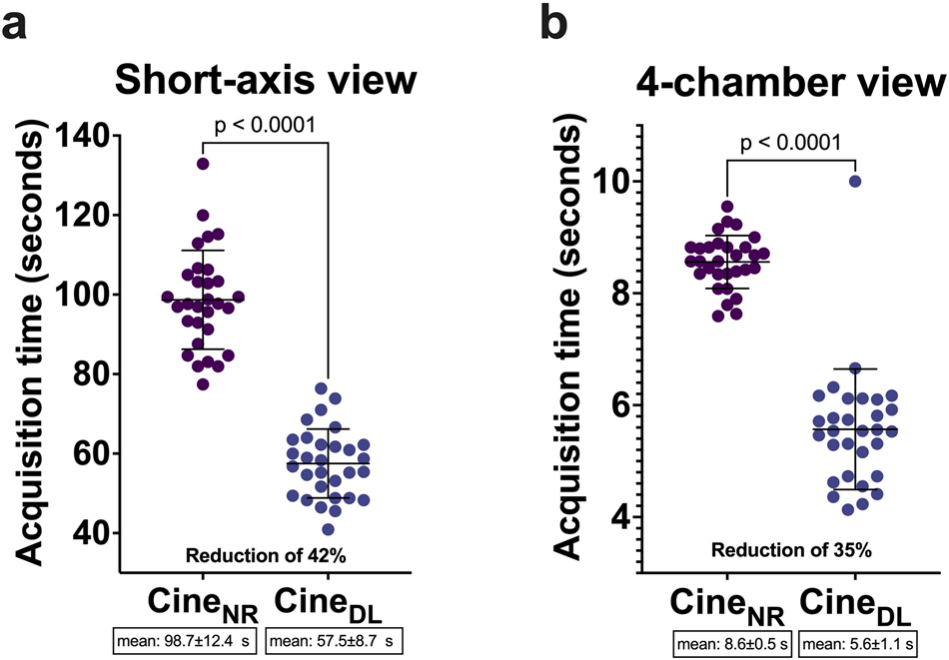
Scatter plots with mean and standard deviation depicting the acquisition times for the normal resolution (cine_NR_) and the low-resolution DL reconstruction (cine_DL_) for short-axis (**a**) and 4-chamber views (**b**)

A significant 35% reduction in acquisition times was observed for cine_DL_ 4-chamber views (5.6 ± 1.1 s vs 8.6 ± 0.5 s; *p* < 0.0001) and 42% for short-axis views (whole stacks 57.5 ± 8.7 s vs 98.7 ± 12.4 s; *p* < 0.0001). A direct comparison of cine_NR_ and cine_DL_ 4-chamber and short-axis images with acquisition times is demonstrated in Fig. 2. Figure 3 visualizes the statistical differences between acquisition times regarding cine_NR_ and cine_DL_ sequences.

### Objective image analysis results

**Fig. 4:**
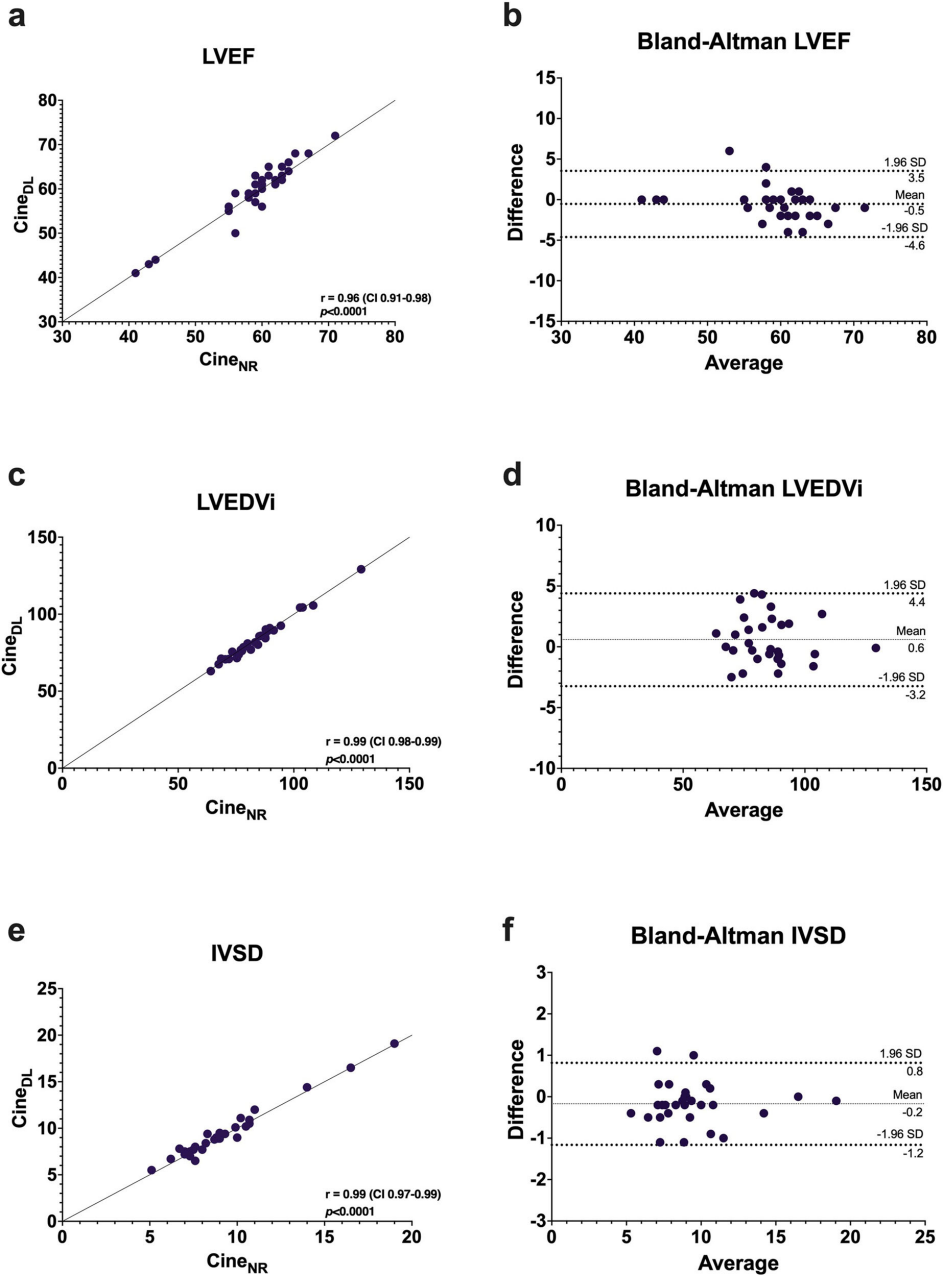
Pearson’s correlation (**a**, **c**, and **e**) and Bland–Altman plots (**b**, **d**, and **f**) comparing LVEF (**a**, **b**), left ventricular LVEDVi (**c**, **d**), and IVSD (**e**, **f**). There was a strong correlation and agreement between normal resolution (cine_NR_) and DL upscaled (cine_DL_) reconstructions

Volumetric analysis yielded no statistically significant differences between the cine_NR_ and cine_DL_ reconstructions (e.g. LVEF (59 ± 7 vs 59 ± 7%; *p* = 0.17). In addition, there was a high level of correlation and excellent agreement for all parameters (e.g. LVEF: *r*: 0.96; mean bias: −0.5; limits of agreement: −4.6, 3.5; ICC: 0.98 [95% confidence interval (CI): 0.94, 0.99]). Volumetry and strain results are summarized in Fig. 4 and Table 2.

**Table 2:** Cardiac volumetry and strain CMR cine normal resolution compared to DL SR reconstructions

Variables	Cine_NR_	Cine_DL_	*p* value^a^	*r*^b^	Mean Bias^c^	LoA^c^	ICC^d^
LVEF, (%)	58.8 ± 6.6	59.3 ± 7.2	0.17	0.96	−0.5	−4.6, 3.5	0.98 (0.94, 0.99)
LVEDVi, (mL/m^2^)	85.0 ± 13.5	84.4 ± 13.7	0.12	0.99	0.6	−3.2, 4.4	0.99 (0.98, 0.99)
IVSD, (mm)	9.3 ± 2.9	9.5 ± 2.9	0.07	0.99	−0.2	−1.2, 0.8	0.99 (0.98, 0.99)
GLS, (%)	−19.5 ± 4.3	−19.8 ± 3.9	0.52	0.89	0.2	−3.5, 4.0	0.94 (0.88, 0.97)
GCS, (%)	−23.2 ± 4.1	−22.6 ± 4.1	0.22	0.78	−0.6	−5.9, 4.7	0.88 (0.74, 0.94)
GRS, (%)	75.5 ± 18.1	71.9 ± 15.3	0.74	0.82	0.6	−19.6, 20.9	0.90 (0.78, 0.95)

*Cine*_NR_ normal-resolution cine sequence, *Cine*_DL_ DL-reconstructed cine sequence, *LVEF* left ventricular ejection fraction, *LVEDVi* left ventricular end-diastolic volume index, *IVSD* interventricular septum thickness at diastole, *GLS* global longitudinal strain, *GCS* global circumferential strain, *GRS* global radial strain

^a^ Paired Student’s *t*-test

^b^ Pearson’s *r*

^c^ Bland–Altmann means bias and limits of agreement (LoA)

^d^ ICC with 95% confidence intervals in brackets

### Subjective image analysis results

**Fig. 5:**
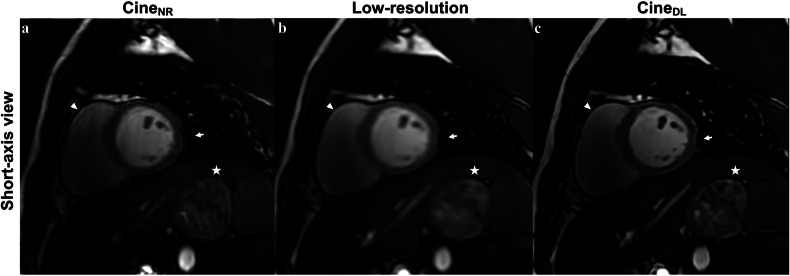
Short-axis view of a 19-year-old male with suspected myocarditis acquired using standard bSSFP normal-resolution (cine_NR_) acquisitions (**a**), low-resolution acquisitions (**b**), and DL SR reconstructions (cine_DL_) (**c**). Cine_DL_ successfully removed the horizontal banding artifact from the cine_NR_ acquisition (star). The left ventricular midventricular antero-/inferolateral epicardial edge (arrow) is more defined in the cine_NR_ acquisition with discernable vessels compared to the low-resolution acquisition, possibly due to the reconstruction algorithm or alternatively due to a small change in slice positioning between the cine_NR_ and low-resolution acquisitions. Additionally, note the more defined sharpening of the lateral wall of the right ventricle (arrowhead) in the cine_DL_ reconstruction

**Fig. 6:**
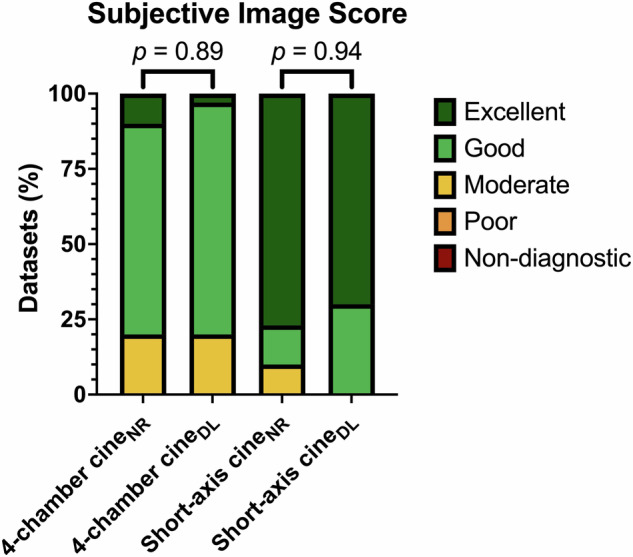
Stacked bar charts showing the distribution of Likert scale scores for subjective image quality for 4-chamber and short-axis normal resolution (cine_NR_) and DL SR (cine_DL_) reconstructions

Edge definition was observed to be slightly better on 4-chamber cine_DL_ views compared to cine_NR_ (median 4.5 [4.0, 5.0] vs 4.0 [3.9, 4.1]; *p* = 0.03, respectively), and fewer artifacts were noted on cine_NR_ 4-chamber views compared to cine_DL_ (median 4.5 [4.0, 5.0] vs 4.0 [4.0, 4.5]; *p* = 0.01, respectively). Otherwise, no significant differences regarding subjective image quality were noted between the sequences. Results are summarized in Table 3. Figure 5 demonstrates differences such as aggressive smoothing/blurring and artifact reduction in short-axis views for the cine_NR_ and cine_DL_ reconstructions. Figure 6 shows the subjective image scores.

**Table 3:** Objective and subjective image quality findings

Variables	Cine_NR_	Cine_DL_	*p* value
Objective image quality			
4-chamber view			
aSNR	31 [26, 57]	29 [22, 48]	0.26
aCNR	21 [15, 38]	20 [13, 32]	0.14
Short-axis view			
aSNR	72 [48, 103]	62 [42, 89]	0.26
aCNR	46 [31, 69]	41 [26, 54]	0.28
Subjective image quality			
4-chamber view			
Contrast	5.0 [4.0–5.0]	4.8 [4.0–5.0]	0.72
Edge definition	4.0 [3.9–4.1]	4.5 [4.0–5.0]	0.03
Artifacts	4.5 [4.0–5.0]	4.0 [4.0–4.5]	0.01
Total	4.4 [4.0–4.7]	4.5 [4.1–4.7]	0.89
Short-axis view			
Contrast	5.0 [5.0–5.0]	5.0 [5.0–5.0]	0.99
Edge definition	5.0 [5.0–5.0]	5.0 [5.0–5.0]	0.65
Artifacts	5.0 [5.0–5.0]	5.0 [5.0–5.0]	0.53
Total	5.0 [4.9–5.0]	5.0 [4.7–5.0]	0.97

All values are given as median with IQR. Wilcoxon matched-pairs signed rank test unless otherwise noted

*aSNR* apparent signal-to-noise ratio, *aCNR* apparent contrast to noise ratio, *Cine*_NR_ normal-resolution cine sequence, *Cine*_DL_ DL-reconstructed cine sequence

### Inter-rater agreement

Inter-rater agreement for subjective image quality was excellent for 4-chamber cine_NR_ (ICC: 0.94 [95% CI: 0.85, 0.97]), 4-chamber cine_DL_ (ICC: 0.90 [95% CI: 0.77, 0.95]), and short-axis cine_NR_ views (ICC: 0.98 [95% CI: 0.96, 0.99]). A good agreement was observed for short-axis cine_DL_ views (ICC: 0.87 [95% CI: 0.74, 0.94]).

### Subgroup analysis

A subgroup analysis was carried out by splitting the study population into a volunteer group (*n* = 20) and a patient group (*n* = 10). No differences between cine_NR_ and cine_DL_ were observed for all volumetric and strain data except IVSD in the patient group (cine_NR_: 10.9 ± 4.2 mm, cine_DL_: 11.1 ± 4.2 mm, *p* = 0.04). All other parameters (LVEF, LVEDVi, global longitudinal strain, global circumferential strain, and global radial strain) demonstrated *p* values above 0.05 with ICC ranging from 0.81 to 0.99 and 0.91 to 0.99 for the volunteer and patient groups respectively. A summary of these results can be found in the Supplement Table S1. A comparison of subjective image quality is found in Table S2. Similar tendencies as the overall comparison was noted for the subgroup analysis regarding subjective image quality, with significant differences observed only regarding edge definition in the patient group on 4-chamber views (cine_NR_: 4.0 [3.4, 4.3]; cine_DL_: 4.8 [4.0, 5.0]; *p* = 0.047) and the volunteer group for artifacts on short-axis views (cine_NR_: 4.5 [4.0, 5.0]; cine_DL_: 4.0 [4.0, 4.0]; *p* = 0.02).

## Discussion

This prospective CMR study evaluated the utility of a DL SR algorithm for the reconstruction of low-resolution bSSFP cine acquisitions and compared them to cine_NR_ acquisitions. Cine_DL_ images were faster to acquire leading to shorter breath holds and did not demonstrate differences regarding image quality, volumetry, or myocardial strain compared to cine_NR_.

CMR combines the increased objectivity and reproducibility of CT with the non-ionizing nature of echocardiography but is constrained by high costs and long examination times, leading to long wait times for patients. Advances in DL technology have the potential to speed up CMR examinations by decreasing acquisition and reconstruction times leading to shorter wait times for patients and allowing a higher throughput of patients per machine. The CMR imaging workflow consists of two processes: planning and acquisition. Both processes require time and are potential targets for scan time reduction. Previous publications have explored possibilities of artificial intelligence-assisted planning and shimming leading to an overall reduction in scan times of approximately 5–13% [26, 27]. Most of the examination time, though, consists of image acquisition and reconstruction. Mathematical equations have traditionally been applied to perform MR image reconstructions with techniques such as SENSE (SENSitivity Encoding), a vendor-specific technique for parallel imaging, and the more recent Compressed SENSE to further accelerate this process by measuring fewer Fourier coefficients. Recently, CNNs have been developed to accelerate MRI reconstructions even further [18]. Acquiring low-resolution images and upscaling them using a DL SR algorithm led to a reduction in overall scan times by an average of 42% and thus shortened breath holds (for short-axis views), in line with other studies using machine learning for accelerated image acquisition achieving reductions in scan times of 45% for brain imaging [28]. Shorter scan times can have a positive psychological effect on patients, increasing compliance and the likelihood of undergoing follow-up examinations. Additionally, a decrease in breath-hold durations required for cine acquisition can have a beneficial effect on image quality, especially in patients with difficulty performing longer breath holds [3]. Lastly, a reduction in acquisition times leads to increased patient throughput, allowing more patients to receive important MRI scans than would otherwise be possible with the limited number of scanners available [29]. Data validating prospectively acquired DL reconstructions comparing them to conventional reconstruction methods is scarce, with a recent meta-analysis calling for the clinical evaluation of subjective image quality and measurement of clinical metrics [30]. This study demonstrates that image quality and volumetric data in complex cine CMR imaging are handled robustly by the DL SR reconstructions. Our use of DL CNNs led to comparable subjective image quality, aSNR, and aCNR without significant differences between cine_NR_ and cine_DL_, in line with other current publications [31, 32]. Bischoff et al [14] were even able to demonstrate better image quality compared to conventional reconstruction methods. Manually derived cardiac function parameters such as LVEF or IVSD measurements showed slightly larger values on cine_DL_ compared to cine_NR_ acquisitions but were not significant and well within their respective standard deviations. Similarly, automated feature tracking software for myocardial strain, a sensitive parameter for cardiac dysfunction, did not measure any significant differences between the volunteer and patient groups in the subgroup analysis. Global circumferential and radial strain demonstrated wider ICC 95% CIs than longitudinal strain, an expected finding as longitudinal strain has been documented to be the most reproducible and robust strain metric. More interestingly, global circumferential and radial strain 95% confidence ranges were wider in the volunteer group than in the patient group. The ability of DL SR to consistently produce results in line with the reference standard in volunteers and even in patients with cardiovascular pathologies, highlights the general applicability of this technique, although larger studies are needed to confirm consistency in patient cohorts. No instances of information loss or confabulation were noticed on assessment, although that was not the main objective of this study. In the past, single-shot CMR acquisitions have been used for the reduction of motion artifacts but are limited by their spatial and temporal resolution. DL algorithms, such as the one employed in this paper provide a fully retrospectively gated alternative with a high spatial and temporal resolution with the ability to shorten acquisition times in order to remove motion artifacts in patients with reduced breath-hold capacities. Kim et al demonstrated that the removal of banding artifacts from bSSFP sequences was possible in an animal model using neural networks [33] while other DL algorithms were successful in removing streaking artifacts [34]. Future applications of artificial intelligence may even provide retrospective motion correction as demonstrated by a generative adversarial network by Kuestner et al [35].

Our study is limited by the small study cohort. For the subjective image quality analysis, raters were blinded to the sequence and sequences were presented in a random order, but due to the nature of the reconstructions, readers were able to distinguish the cine_NR_ from the cine_DL_ reconstructions in most cases. The quality of DL neural networks is highly dependent on the training data they receive and may play a central role in image output quality [36]. Currently, available networks must be trained on specific patterns and anatomical variances for each disease for it to be effective. While we did not observe any loss of data or blurring in our study, we also did not specifically look at this as would be the case in clinical studies, for example looking to detect small brain tumors. Furthermore, low-resolution acquisitions for DL reconstruction were acquired after cine_NR_ acquisitions which consisted of up to 14 breath-holds, leading to a nonequal starting condition of the low-resolution acquisitions compared to the cine_NR_ acquisitions.

In conclusion, SR DL reconstruction of CMR cine sequences acquired with a lower spatial resolution led to a significant reduction in acquisition times of 35–42% on average and shorter breath hold durations without a significant difference in volumetric results or subjective image quality in healthy volunteer as well as patients.

## Supplementary information

ELECTRONIC SUPPLEMENTARY MATERIAL

## Abbreviations

aCNR: Apparent contrast-to-noise ratio
aSNR: Apparent signal-to-noise ratio
bSSFP: Balanced steady-state free precession
Cine_DL_: Deep learning reconstructed cine sequence
Cine_NR_: Normal-resolution cine sequence
CMR: Cardiovascular magnetic resonance
CNN: Convolutional neural network
Compressed SENSE: Compressed sensitivity encoding
DL: Deep learning
ICC: Intraclass correlation coefficient
IQR: Interquartile range
IVSD: Interventricular septum thickness at diastole
LVEDVi: Left ventricular end-diastolic volume index
LVEF: Left ventricular end-diastolic volume
SR: Super-resolution
