# Supplementary material for: Deep learning super-resolution reconstruction for fast and high-quality cine cardiovascular magnetic resonance
Source: Eur Radiol. 2024 Oct 23;35(5):2877–87. doi: 10.1007/s00330-024-11145-0 (PMC12021735; doi:10.1007/s00330-024-11145-0)
Supplement: Supplementary file 1 — ELECTRONIC SUPPLEMENTARY MATERIAL [file 330_2024_11145_MOESM1_ESM.pdf]

Deep learning super-resolution reconstruction for fast and high-quality cine cardiovascular magnetic resonance

ELECTRONIC SUPPLEMENTARY MATERIAL

Table S1. Volumetry and strain comparing cardiovascular magnetic resonance cine normal resolution and deep learning super-resolution reconstructions.

	Volunteers (n=20)							Patients (n=10)						
**Variables**	**$Cine_{NR}$**	**$Cine_{DL}$**	***p* value[a]**	**r[b]**	**Mean Bias[c]**	**LoA[c]**	**ICC[d]**	**$Cine_{NR}$**	**$Cine_{DL}$**	***p* value[a]**	**r[b]**	**Mean Bias[c]**	**LoA[c]**	**ICC[d]**
LVEF (%)	60.0±3.2	60.3±4.1	0.57	0.83	-0.3	-4.8, 4.2	0.90 (0.74, 0.96)	56.3±10.3	57.3±11.1	0.07	0.99	-1.0	-4.1, 2.1	0.99 (0.97, 0.99)
LVEDVi (ml/m^2)	85.4±10.9	84.7±10.9	0.21	0.98	0.7	-3.6, 5.0	0.99 (0.97, 0.99)	84.0±18.3	83.7±18.7	0.33	0.99	0.4	-2.4, 3.1	0.99 (0.99, 0.99)
IVSD (mm)	8.5±1.6	8.7±1.5	0.35	0.94	-0.1	-1.2, 1.0	0.97 (0.92, 0.99)	10.9±4.2	11.1±4.2	0.04	0.99	-0.3	-1.0, 0.4	0.99 (0.98, 0.99)
GLS (%)	-20.4±3.8	-20.5±3.8	0.94	0.87	0.03	-3.7, 3.8	0.93 (0.83, 0.97)	-17.7±4.8	-18.4±4.0	0.33	0.92	0.6	-3.2, 4.4	0.95 (0.80, 0.99)
GCS (%)	-23.8±2.8	-23.3±4.0	0.47	0.72	-0.5	-5.9, 5.0	0.81 (0.52, 0.93)	-22.1±5.9	-21.1±4.2	0.31	0.92	-0.9	-6.2, 4.3	0.93 (0.72, 0.98)
GRS (%)	79.3±12.1	75.6±11.7	0.07	0.73	3.7	-13.4, 20.7	0.83 (0.57, 0.93)	58.9±21.1	64.4±19.3	0.15	0.85	-5.4	-27.1, 16.2	0.91 (0.65, 0.98)
Acquisition times (s)	95.7±10.9	55.9±8.1	<0.0001	0.46	39.8	20.0, 59.7	0.09 (-0.05, 0.36)	104.7±13.6	60.8±9.3	<0.0001	.81	43.9	27.8, 60.0	0.17 (-0.33, 0.60)

[a] paired Student's t test. [b] Pearson's r. [c] Bland-Altmann mean bias and limits of agreement (LoA). [d] Intraclass correlation coefficient (ICC) with 95% confidence intervals in brackets.
$Cine_{NR}$: normal-resolution cine sequence. $Cine_{DL}$: DL-reconstructed cine sequence. LVEF: left ventricular ejection fraction. LVEDVi: left ventricular end diastolic volume index. IVSD: interventricular septum thickness at diastole. GLS: global longitudinal strain. GCS: Global circumferential strain. GRS: Global radial strain.

Table S2. Subgroup analysis of objective and subjective image quality findings between volunteers and patients.

	Volunteers (n=20)			Patients (n=10)		
**Variables**	**$Cine_{NR}$**	**$Cine_{DL}$**	***p***	**$Cine_{NR}$**	**$Cine_{DL}$**	***p***
			**Objective image quality**			
**4-chamber view**						
aSNR	29 [23, 50]	24 [19, 34]	0.44	49 [32, 70]	47 [29, 73]	0.49
aCNR	18 [14, 30]	15 [11, 23]	0.27	33 [21, 46]	33 [19, 46]	0.36
**Short-axis view**						
aSNR	66 [35, 110]	65 [40, 85]	0.49	93 [57, 101]	60 [42, 99]	0.15
aCNR	43 [25, 76]	41 [23, 60]	0.50	58 [42, 66]	41 [26, 59]	0.17
			**Subjective image quality**			
**4-chamber view**						
Contrast	5.0 [4.5, 5.0]	4.0 [4.0, 5.0]	0.18	4.8 [4.0, 5.0]	5.0 [4.8, 5.0]	0.38
Edge definition	4.0 [4.0, 4.4]	4.3 [3.6, 5.0]	0.38	4.0 [3.4, 4.3]	4.8 [4.0, 5.0]	**0.047**
Artefacts	4.5 [4.0, 5.0]	4.0 [4.0, 4.0]	**0.02**	4.5 [4.0, 5.0]	4.3 [4.0, 4.6]	0.22
Total	4.5 [3.9, 4.7]	4.4 [3.8, 4.7]	0.31	4.3 [4.0, 4.7]	4.5 [4.3, 4.7]	0.10
**Short-axis view**						
Contrast	5.0 [5.0, 5.0]	5.0 [5.0, 5.0]	1.00	5.0 [4.9, 5.0]	5.0 [5.0, 5.0]	0.50
Edge definition	5.0 [5.0, 5.0]	5.0 [5.0, 5.0]	0.99	5.0 [4.1, 5.0]	5.0 [4.9, 5.0]	0.50
Artefacts	5.0 [5.0, 5.0]	5.0 [4.6, 5.0]	0.75	5.0 [4.5, 5.0]	5.0 [5.0, 5.0]	0.50

Total	5.0 [5.0, 5.0]	5.0 [4.7, 5.0]	0.59	5.0 [4.5, 5.0]	5.0 [4.7, 5.0]	0.44

All values are given as median with interquartile range. Wilcoxon matched-pairs signed rank test unless otherwise noted. aSNR: apparent signal to noise ratio. aCNR: apparent contrast to noise ratio. Cine$_{NR}$: normal-resolution cine sequence. Cine$_{DL}$: DL-reconstructed cine sequence.
